# Altitude impact on the chemical profile and biological activities of *Satureja thymbra* L. essential oil

**DOI:** 10.1186/s12906-020-02982-9

**Published:** 2020-06-12

**Authors:** Noha Khalil, Lamya El-Jalel, Miriam Yousif, Mariam Gonaid

**Affiliations:** 1 Faculty of Pharmaceutical Sciences and Pharmaceutical Industries, Future University in Egypt, Cairo, 11835 Egypt; 2 Faculty of Natural Resources and Environmental Sciences, Omar Al-Moukhtar University, 991, Albayda, Libya

**Keywords:** Altitude, Anthelmintic, Antimicrobial, Cytotoxic, Essential oil, *Satureja*

## Abstract

**Background:**

Several agricultural or environmental factors affect plants’ chemical and pharmacological properties.

**Methods:**

In this study, the essential oil of Libyan *Satureja thymbra* was isolated from plants collected during two successive years at two different altitudes; Wasita (WEO) and Safsaf (SEO), 156 and 661 m above sea level, respectively.

**Results:**

GC/MS allowed the identification of 21 and 23 compounds, respectively. Thymol prevailed in WEO (26.69%), while carvacrol prevailed in SEO (14.30%). Antimicrobial activity was tested by agar-well diffusion method, and MIC/MLC values were determined by broth dilution method. Values of MIC/MLC were 0.125/0.25 μg/ml for SEO against *S. aureus*, *P. mirabilis* and *K. pneumonia* and for WEO against *B. subtilus*. It was observed that plants growing at lower altitude in Wasita locality had better antifungal activity, while those growing at higher altitude at Safsaf locality had better antibacterial activity. Both essential oils had a better anthelmintic activity than the standard piperazine citrate against a tested earthworm. However, SEO oil had a significantly higher anthelmintic activity than WEO. Cytotoxicity of the oils tested using SRB assay on human breast cancer (MCF-7) and colon cancer cell lines (HCT-116) showed better activity for SEO, especially against HCT-116 with IC_50_ 2.45 ± 0.21 μg/ml.

**Conclusions:**

Thus, altitude is an important factor that should be considered as it affected the yield, composition and biology of the plant extracts.

## Background

The Libyan flora is spread over a diversity of geomorphologic regions which in return gives rise to a variety of climatic conditions suitable for the growth of different types of plants [1]. One of the widespread plants in Al Jabal Al Akhdar region in Libya are members of family Lamiaceae like *Satureja* genus [2]. *Satureja* comprises more than 200 species of popular herbs or shrubs that are often aromatic [3]. In Al Jabal Al Akhdar, two main species of *Satureja* dominate, *S. thymbra* and *S. fortii* [4]. *Satureja thymbra* L.*,* known as pink savory, is an aromatic plant with oregano-like smell that is endemic to the Mediterranean region. The main chemical classes identified in the essential oil include monoterpene hydrocarbons such as *p*-cymene and γ-terpinene, oxygenated monoterpenes, especially phenolic constituents as thymol and carvacrol. Also, sesquiterpene hydrocarbons were detected as β-caryophyllene and α-humulene as well as oxygenated sesquiterpenes such as caryophyllene oxide [5–9]. The plant is also rich in other phenolic constituents like flavonoids and flavonoid glycosides as well as tannins, acids and exudates [10]. Due to the presence of these compounds, *S. thymbra* L. has been evaluated as antibacterial, antifungal and antiviral agent [11]. *The essential* oil proved an antimicrobial activity against several bacteria and fungi like *Escherichia coli*, *Pseudomonas aeruginosa*, *Salmonella typhimurium*, *Shigella sonnei*, *Staphylococcus aureus* and some resistant strains such as *Stenotrophomonas maltophilia*, *S. maltophilia*, and *Chryseomonas luteolaas* as well as the yeast *Candida albicans* [8, 12–15]. The essential oil of *S. thymbra* (1%) has also been reported to possess a strong bactericidal activity against bacterial biofilms formed on stainless steel by some pathogenic bacteria [16]. The oil has also shown an in-vitro inhibitory activity against SARS-CoV and HSV-1 replication by visually scoring of the virus-induced cytopathogenic effects post-infection with a high selectivity index (SI) against HSV-1 [17]. The oil also had a high SI when tested for its cytotoxicity in Vero cells using MTT assay [18]. Essential oil of *S. thymbra* has also been reported to possess an insecticidal activity against the mosquito *Culex pipiens* biotype *molestus*, acaricidal effect against *Hyalomma marginatum*, genotoxic activity against *Drosophila* as well as insecticidal activity against three stored-product insects [19–22]. Other reported activities for the oil included improving circulation and thus being used in the treatment of arthritis, rheumatism, as a wound healing promoter and in painful joints and muscles [23]. Being evaluated for cholinesterase inhibitory and antioxidative effects, the essential oil is suggested to treat amnesia and Alzeheimer’s diseases [24]. Traditionally, it has been used in food flavoring, perfumery and in local home remedies as antiseptic and diuretic [25].

Plants’ essential oil content and composition, and in-turn, its biological activity, has proved to be related to several factors including altitude, genetic, climate, topography, genotype, growing conditions and harvest time. Also, different chemotypes have been described for a number of medicinal plants [26–29]. Moreover, studies have shown that the different characteristics of medicinal plant may be affected by a variety of ecological factors such soil composition, temperature, humidity and other climatic conditions [30–33]. Consequently, these changes in content and composition would definitely affect the pharmacological activity of the essential oil. Specifically, several studies show that chemical metabolic profile of Lamiaceae plants is strongly affected by environmental conditions such as geographical cultivation area, cultivation and harvesting period as well as local ecological conditions [34]. For example, altitude is one of the factors that affect plant metabolism [35]. Previous reports revealed that phenol-rich Lamiaceae species have shown that the great diversity in the content of thymol and carvacrol in the essential oils is associated with climatic factors [36, 37]. Altitude affected the quantity and quality of the essential oil of several studied plants like *Thymus kotschyanus* (Lamiaceae), *Teucrium hyrcanicum* (Lamiaceae), *Cymbopogon olivieri* (Poaceae), *Mentha piperita* (Lamiaceae), *Tanacetum polycephalum* (Asteraceae), *Ziziphora clinopodioides* (Lamiaceae) and *Lavandula angustifolia* (Lamiaceae) [33, 34, 38]. To further investigate the effect of altitude on the Libyan-growing medicinal plant; *Satureja thymbra*, the present work aimed at studying the impact of two different altitudes (Wasita and Safsaf in Al Jabal Al Akhdar region in Libya), on the chemical profile, antimicrobial, anthelmintic and cytotoxic activity of the essential oils of *S. thymbra* aerial parts collected during fall 2017 and 2018.

## Methods

### Plant material

Samples of aerial parts of *S. thymbra* were obtained from two different localities (50 plants from random locations in each locality), during fall 2017 and fall 2018 from plants in Al-Jabal Al-Akhdar in Libya, from Wasita locality (32°52′30.68″N, 13°11′14.86″E) at 150 m above sea level and from Safsaf locality (32°78′19.51″N, 21°95′23.92″E) at 661 m above sea level. The plant is not endangered, so no permission was needed for its collection. Plants identity was authenticated by staff members of the Plant Taxonomy Department of the Faculty of Science at Omar Al Mokhtar University, Al Bydaa, Lybia. Voucher specimens were deposited at the Faculty of Pharmacy, Omar Al-Moukhtar University (STTB-8 and STTB-9). Plants were dried in shade, powdered and refrigerated in closed containers until use.

### Isolation of essential oils

Samples (100 g of dried plant in 500 ml distilled water), were separately hydro-distilled with a Clevenger apparatus for 4 h. Percentage oil yield was calculated/dry weight using the eq. (W1/W2 × 100, where W1 is the weight if the oil in grams and W2 is the total weight of dried plant used). The obtained essential oil was dried over anhydrous sodium sulfate and kept refrigerated in sealed amber vials till analysis. Specific gravity of the obtained essential oils was determined according to the Egyptian Pharmacopoeia (2005).

### GC/MS analysis of the essential oil samples

An Agilent 7890A gas chromatograph (Agilent Technologies, Palo Alto, CA, USA) with a capillary column RTX-5MS (30 m × 0.32 mm, film thickness 0.25 μm) was used for the GC/MS analysis of the essential oils. This was coupled to an Agilent 5975C mass selective detector. The initial oven temperature was 40 °C for 2 min, then it was raised at the rate of 5 °C/min until it reached 210°. The injector and detector temperatures were 290 and 300 °C, respectively. Helium carrier gas was used at a flow rate of 2 ml/min. Manual split mode injection was applied (0.1 μl, each). EI mode was used for recording the mass spectra. The range for m/z was 35–500. Ionization voltage was 70 eV and ion source temperature was set at 230 °C. The above conditions were applied for the analysis of a homologous series of n-alkanes to calculate retention index (RI). Identification was based on comparison of KI with literature [39], in addition to obtained data from Wiley’s MS libraries. Authentic compounds (Sigma-Aldrich, Germany) were also used for identification of some compounds (Table 2).

### Gas chromatography/flame ionization detection (GC-FID)

The GC analyses were carried out on a Focus GC® (Thermo fisher scientific®, Milan, Italy) equipped with TR5-MS fused bonded column (30 m × 0.25 mm × 0.25 μm) (Thermo fisher scientific®, Florida, USA) and FID detector; carrier gas was nitrogen (1.5 ml/ min); the operating conditions were: initial temperature 40 °C, 1 min. Isothermal followed by linear temperature increase till 230 °C at a rate of 4 °C / min. 230 °C, then 5 min. Isothermal. Detector and injector temperatures were 300 and 220 °C, respectively. The split ratio was 1: 20. Chrom-card® chromatography data system ver. 2.3.3 (Thermo Electron Corp.®, Florida, USA) was used for recording and integrating of the chromatograms. Average areas under the peaks of three independent chromatographic runs were used for calculation the % composition of each component.

### Antimicrobial activity of the essential oils

#### Source of microbial cultures

Standard reference strains (American Type Culture Collection “ATCC” for bacteria and fungi) were used for assessing the antimicrobial activity of the essential oils. Gram positive bacteria: *Staphylococcus aureus* (ATCC 6538), *Bacillus subtilis* (ATCC 6051). Gram negative bacteria: *Escherichia coli* (ATCC 8739), *Pseudomonas aeruginosa* (ATCC 9027), *Proteus mirabilis* (ATCC 7002), *Klebsiella pneumoniae* (ATCC 13883). Fungal microorganisms: *Candida albicans* (ATCC 10231), *Aspergillus niger* (ATCC 16888). Microbial inoculate of bacterial and fungal cultures were prepared as suspensions in Roux bottles using Trypticase soy agar (TSA) and Sabouraud dextrose agar (SDA) media according to the directions of the manufacturer (Sigma, USA).

#### Agar-well diffusion method

*Satureja thymbra* essential oils were prepared as an emulsion (10% w/w) using Tween 80. Sterilization of the obtained emulsion was achieved using a 0.45 μm membrane filter. Agar-well diffusion method was performed according to the Clinical and Laboratory Standards Institute [40]. The resultant inhibition zones were measured in mm, and the average values were taken. Ampicillin, Ciprofloxacin and Amphotericin B were used as standard antimicrobial agents. A blank prepared from the same concentration of Tween 80 was included.

#### Determination of minimum inhibitory and lethal concentrations (MIC/MLC) by broth dilution method

Broth dilution method was used for determination of minimum inhibitory and minimum lethal concentrations of the oil (MIC/MLC) against the previously mentioned standard microorganisms [40]. Determination was achieved on 96 well culture plates using a microorganism suspension at a density of 10^5^ CFU/ml with Casein Soy Broth (CSB) incubated for 24 h at 37 °C for bacteria, and Sabouraud Dextrose Broth (SDB) incubated for 48 h at 25 °C for yeasts. Serial dilutions of essential oils (0.125–5 μl/ml) were prepared with Brain Heart infusion (BHI) broth medium in test tube and mixed with bacterial suspensions to give a volume of 4 ml and a final concentration of bacteria of approximately 5 × 10^4^ CFU/ml. Final solutions were incubated at the temperatures mentioned earlier. The MIC is the lowest concentration which gave no visible growth, while the MLC was determined by sub-culturing 100 *μ*l from each negative test tube onto plate count agar (PCA) plates. MLC was defined as the lowest concentration resulting in a negative subculture or giving presence of only one colony after incubation. Negative controls lacking the oil were assayed simultaneously. The experiments were carried out in three replicates.

### Anthelmintic activity of the essential oils

*Allolobophora caliginosa;* an adult earthworm, was used to evaluate the anthelmintic activity of the essential oils at different concentrations. To obtain different concentrations of the essential oil (1, 2 and 3% v/v), 3% essential oil was diluted in 1% aqueous tween 80. Anthelmintic assay was carried out by dividing the earth worms into three groups each containing 6 worms (each worm ≥10 cm long). A control containing 3% aqueous tween 80 was used. The reference anthelmintic drug piperazine citrate (Sigma-Aldrich, USA) was also prepared in 0.1% solution using tween 80. Time of paralysis was recorded when no movement was observed, while time of death when the worms didn’t move after being dipped in warm water (50 °C) [41].

### In-vitro screening of cytotoxic activity

#### Human tumor cell lines

The HCT-116 (human colon carcinoma) cell lines and MCF-7 (human breast adenocarcinoma) cells, maintained in the laboratory of Cancer Biology Department of National Cancer Institute, Cairo, Egypt (obtained originally from Sigma-Aldrich, Germany) were used for cytotoxicity assay. Normal hamster lung fibroblasts (V79 cells) were included as control.

#### Cytotoxicity assay

The essential oils at different concentrations (0–10 μg/ml in 2% DMSO) were tested for cytotoxicity against the fore mentioned human tumor cell lines adopting sulforhodamine B stain (SRB) assay [42]. The relation between survivals and the oil concentration was plotted to get the survival curve of each tumor cell line after the application of specific concentration. The results were compared to those of the standard cytotoxic drug; doxorubicin (10 mg adriamycin hydrochloride, in 5 ml IV injection, Pharmacia, Italy) at the same concentration was used as standard anti-tumor. The dose of the test solutions which reduces survivals to 50% (IC_50_) was calculated as well as selectivity index. A blank prepared from the same concentration of Tween 80 was included.

### Data analysis

Tests were conducted in triplicate and values recorded as mean ± SEM. Results were analyzed by GraphPad Prism® v.5 software. Significant differences among means of different samples were analyzed using paired*-t-*test at *p* ≤ 0.05.

## Results

### Effect of altitude on essential oil physical properties, yield and composition

Both Wasita essential oil (WEO) and Safsaf essential oil (SEO) were obtained in yellow color with a strong aromatic odor. During fall 2017, WEO had a significantly higher yield and specific gravity (1.6 ± 0.03%v/w, 0.9 ± 0.04, respectively, *p* < 0.01, paired*-t-*test) than SEO (1.4 ± 0.02%v/w and 0.6 ± 0.02, respectively). No significant difference in the results was observed during fall 2018 than those obtained during fall 2017 (Table 1).

**Table 1:** Physical properties and percentage yield of *S. thymbra* essential oils obtained from Wasita (WEO) and Safsaf (SEO) localities during two successive years

Locality	% EO (w/v)	Specific gravity of EO	Color of EO	Odor of EO
Wasita W1	1.60 ± 0.03^a^	0.90 ± 0.04^a^	Yellow	Strong aromatic
Wasita W2	1.55 ± 0.05^a^	0.90 ± 0.08^a^		
Safsaf S1	1.44 ± 0.02^b^	0.60 ± 0.02^b^		
Safsaf S2	1.38 ± 0.04^b^	0.60 ± 0.06^b^		

Values are ±SEM (*n* = 5), different letters in same column denote significant difference at p < 0.01, paired*-t-*test

W1 and W2 are plants collected from Wasita during fall 2017 and 2018, respectively. S1 and S2 are plants collected from Safsaf during fall 2017 and 2018, respectively

GC/MS analysis of WEO and SEO allowed the identification of 21 and 23 compounds, representing 88.17 and 93.84% in both oils, respectively during fall 2017 (Table 2). Monoterpene hydrocarbons had almost the same percentage in both oils (16.6 and 16.1% in WEO and SEO, respectively) represented mainly by α-thujene and *p*-cymene which had higher percentage in SEO (8.7 and 2.3% in SEO and 3.8 and 7.5% in WEO, respectively). On the other hand, two monoterpene hydrocarbons; α-terpinolene and allo-ocimene were detected in WEO only. Oxygenated monoterpenes was the most prevailing class in both oils (35.6 and 32.1% in WEO and SEO, respectively) represented mainly by the phenolic constituents; thymol and carvacrol. Thymol was the major identified compound in WEO (29.6%), while carvacrol was the major identified compound in SEO (14.3%). Sesquiterpene hydrocarbons were represented mainly by viridiflorine in WEO (3.4%) and δ-cadinene in SEO (6.4%). Oxygenated sesquiterpenes had a much higher percentage in WEO than SEO (27.2 and 2.1%, respectively). Iso-spathulenol was the only oxygenated sesquiterpene in SEO (2.1%) which was also the major oxygenated sesquiterpene detected in WEO (13.4%) among other oxygenated sesquiterpenes present. Other classes of compounds were 27.2% in SEO and only 1.1% in WEO, represented by 2-tetra-butyl-4-methyl-phenol, which was completely absent in SEO. Generally, no significant difference in the results was observed during fall 2018 than those obtained during fall 2017.

**Table 2:** Effect of altitude on the chemical profile of the essential oils of *S. thymbra* essential oils obtained from Wasita (WEO) and Safsaf (SEO) localities during two successive years

Compound	Retention index (RI)		Rel. abundance (%)				Methods of identifications
	Cal.	Reported	Wasita W1 (156 m)	Wasita W2 (156 m)	Safsaf S1 (661 m)	Safsaf S2 (661 m)	
***Monoterpene hydrocarbons***							
α-thujene	918	916	3.82 ± 0.51 ^a^	3.51 ± 0.14 ^a^	4.57 ± 0.21 ^b^	4.69 ± 0.18 ^b^	RI, MS
*p*-cymene	1025	1023	7.50 ± 1.01 ^a^	7.25 ± 0.92 ^a^	8.76 ± 0.21 ^a^	8.12 ± 0.41 ^a^	RI, MS, AT
γ-terpinene	1052	1053	1.38 ± 0.22 ^a^	0.98 ± 0.34 ^a^	2.34 ± 0.78 ^b^	1.96 ± 0.98 ^b^	RI, MS, AT
dehydro-*p*-cymene	1070	1067	–	–	0.50 ± 0.11 ^a^	0.94 ± 0.22 ^b^	RI, MS
α-terpinolene	1079	1082	1.85 ± 0.48 ^a^	1.27 ± 0.33 ^a^	–	–	RI, MS
allo-ocimene	1118	1119	2.08 ± 1.14 ^a^	2.11 ± 0.94 ^a^	–	–	RI, MS, AT
			*16.63*	*15.12*	*16.17*	*15.71*	
***Oxygenated monoterpenes***							
thymol	1291	1299	29.69 ± 3.25 ^a^	28.69 ± 4.12 ^a^	9.19 ± 2.44 ^b^	8.85 ± 3.12 ^b^	RI, MS,AT
carvacrol	1298	1302	2.77 ± 0.25 ^a^	2.54 ± 0.21 ^a^	14.30 ± 2.55 ^b^	14.78 ± 2.12 ^b^	RI, MS, AT
4,5-dimethyl-2-ethyl phenol	1305	–	2.09 ± 0.45^a^	2.28 ± 0.26 ^a^	1.86 ± 0.59 ^b^	1.41 ± 0.45 ^b^	RI, MS
2,3,5,6-tetramethyl phenol	1361	–	1.06 ± 0.58 ^a^	1.06 ± 0.58 ^a^	3.90 ± 1.33 ^b^	3.52 ± 0.97 ^b^	RI, MS
2,3,5,6-tetramethyl,3,4-diethyl phenol	1296	1301	–	–	2.93 ± 0.41 ^a^	3.02 ± 0.44 ^a^	RI, MS
			*35.61*	*34.57*	*32.18*	*31.10*	
***Sesquiterpene hydrocarbons***							
*trans*-β- caryophellene	1412	1410	0.51 ± 0.21 ^a^	0.45 ± 0.19 ^a^	3.57 ± 1.02 ^b^	3.14 ± 1.22 ^b^	RI, MS, AT
aromadendrene	1427	1423	1.42 ± 0.12 ^a^	1.32 ± 0.09 ^a^	1.47 ± 0.33 ^a^	1.78 ± 0.23 ^a^	RI, MS, AT
α-humulene	1441	1438	1.23 ± 0.78 ^a^	0.99 ± 0.95 ^a^	1.58 ± 1.06 ^a^	1.42 ± 0.96 ^a^	RI, MS, AT
viridiflorine	1491	1487	3.40 ± 1.18 ^a^	3.15 ± 1.21 ^a^	–	–	RI, MS, AT
δ-cadinene	1525	1521	1.01 ± 0.77 ^a^	1.41 ± 0.45 ^a^	6.48 ± 2.31 ^b^	6.12 ± 2.96 ^b^	RI, MS, AT
			*7.57*	*7.32*	*13.10*	*12.46*	
***Oxygenated sesquiterpenes***							
α-cedrene oxide	1569	1566	3.14 ± 2.11 ^a^	4.68 ± 1.01 ^a^	–	–	RI, MS, AT
caryophllene oxide	1570	1568	1.41 ± 0.69 ^a^	1.79 ± 0.25 ^a^	–	–	RI, MS, AT
iso spathulenol	1577	1585	13.45 ± 1.37 ^a^	12.45 ± 1.77 ^a^	2.18 ± 1.05 ^b^	2.44 ± 0.91 ^b^	RI, MS, AT
β-oplopenone	1607	1603	0.51 ± 0.33 ^a^	0.49 ± 0.24 ^a^	–	–	RI, MS
caryophyllenol	1645	1648	3.84 ± 2.04 ^a^	3.74 ± 2.47 ^a^	–	–	RI, MS
valerenol	1706	1711	4.85 ± 1.25 ^a^	4.54 ± 1.05 ^a^	–	–	RI, MS
			*27.2*	*26.69*	*2.18*	*2.44*	
***Diterpenes***							
cembrene	1948	1941	–	–	2.97 ± 0.72 ^a^	3.01 ± 0.55 ^a^	RI, MS
***Others***							
2-tetra-butyl-4-methyl-phenol	1513	–	1.16 ± 0.45 ^a^	3.29 ± 0.78 ^b^	–	–	RI, MS
*p*-anisaldehyde	1277	1279	–	–	1.37 ± 0.56 ^a^	2.25 ± 0.77 ^b^	RI, MS, AT
1,2-diethyl-3,4-dimethyl-benzene	1075	1076	–	–	1.22 ± 0.44 ^a^	1.04 ± 0.62 ^a^	RI, MS
ethyl-tetra-methyl-cyclo penta-diene	1082	1085	–	–	4.04 ± 1.47 ^a^	3.94 ± 1.66 ^a^	RI, MS
4, methoxy-6-methyl-2(3′,5′-dimethoxy benzyl) benzoic acid	1685	–	–	–	2.33 ± 1.22 ^a^	1.93 ± 1.36 ^a^	RI, MS
1,2,3,1′,2′,3′-hexamthyl-bicyclopentyl 2,2′-diene	1122	1126	–	–	6.01 ± 2.58 ^a^	5.86 ± 2.98 ^a^	RI, MS
2-hydroxy-3,4,6,7-tetra methoxy phenanthrene	1975	–	–	–	2.01 ± 1.03 ^a^	2.41 ± 0.65 ^a^	RI, MS
1.8-dimethoxy-3-methylanthracene,9,10-dione	2035	–	–	–	4.85 ± 2.66 ^a^	3.24 ± 1.25 ^b^	RI, MS
benzie-α-pyrone	2435	2442	–	–	2.44 ± 0.89 ^a^	2.41 ± 0.41 ^a^	RI, MS
			*1.16*	*3.29*	*27.24*	*23.08*	
*Total number of identified compounds*			*21*	*21*	*23*	*23*	
*Total percentage of identified compounds*			*88.17*	*86.99*	*93.84*	*87.80*	

MS: Identification based on mass spectral data; RI: identification based on retention index relative to standard *n*-alkanes; AT: Identification based on co-chromatography with authentic samples, (−) = not detected. Values are ±SEM (*n* = 3), means followed by different letters in same row denote significant difference at p < 0.05, paired*-t-*test

W1 and W2 are plants collected from Wasita during fall 2017 and 2018, respectively. S1 and S2 are plants collected from Safsaf during fall 2017 and 2018, respectively

### Effect of altitude on antimicrobial activity of the essential oils

Testing of antimicrobial activity of the oils by agar-well diffusion method revealed that highest inhibition zone was observed for SEO collected during fall 2017 (55.7 ± 0.7 mm) against the gram-negative *K. pneumonia*, while the weakest activity was observed for WEO collected during the same season, against *P. aeruginosa* with inhibition zone 11.4 ± 0.2 mm (Table 3). Both oils had significantly (*p* < 0.05, paired*-t-*test) higher activity than ampicillin against the tested gram-positive bacteria. SEO had significantly higher activity than WEO against *B. subtilus* with inhibition zones 49.2 ± 1.1 mm and 39.2 ± 1.5 mm, respectively. However, the difference in activity between both oils against *S. aureus* was not significant. Also, activity of both oils was significantly higher than ciprofloxacin against all tested gram-negative bacteria, except for SEO against *P. aeruginosa*, which had the same activity as ciprofloxacin, while WEO had significantly lower activity than ciprofloxacin. Antibacterial activity of WEO was significantly higher than SEO against *E. coli* and *P. mirabilis*. WEO had significantly higher antifungal activity than SEO; however, both oils’ antifungal activity was significantly higher than the standard amphotericin B against *C. albicans*, but significantly lower than the standard amphotericin B against *A. niger*.

**Table 3:** Effect of altitude on the antimicrobial activities of the essential oils of S. thymbra essential oils obtained from Wasita (WEO) and Safsaf (SEO) localities during two successive years

Test Organisms	Inhibition Zone (mm)					
	Blank	WEO (W1)	WEO (W2)	SEO (S1)	SEO (S2)	Standard
*Gram positive organisms*						Ampicillin
*Staphylococcus aureus* ATCC 6538	(−)	26.2 ± 0.9 ^aB^	25.1 ± 1.0 ^aB^	27.0 ± 0.3 ^aB^	28.0 ± 0.1 ^aB^	12.5 ± 0.5 ^A^
*Bacillus subtilis* ATCC 6051	(−)	39.2 ± 1.5 ^aB^	39.2 ± 1.5 ^aB^	49.2 ± 1.1 ^bB^	49.5 ± 0.6 ^bB^	27.4 ± 0.7 ^A^
*Gram negative organisms*						Ciprofloxacin
*Escherichia coli* ATCC 8739	(−)	46.6 ± 1.2 ^aB^	46.9 ± 0.9 ^aB^	37.2 ± 0.9 ^aB^	38.2 ± 1.2 ^aB^	23.4 ± 0.6 ^A^
*Pseudomonas aeruginosa* ATCC 9027	(−)	11.4 ± 0.2 ^aB^	11.9 ± 0.5 ^aB^	19.8 ± 0.4 ^bB^	18.2 ± 0.3 ^bB^	20.6 ± 1.2 ^A^
*Proteus mirabilis*atcc 7002	(−)	31.6 ± 0.4 ^aB^	30.2 ± 0.4 ^aB^	30.6 ± 0.2 ^bB^	29.8 ± 0.5 ^bB^	19.8 ± 0.6 A
*Klebsiella pneumoniae* ATCC 13883	(−)	55.4 ± 0.2 ^aB^	53.2 ± 0.9 ^aB^	55.7 ± 0.7 ^aB^	56.4 ± 0.2 ^aB^	22.5 ± 0.5 A
*Fungi*						Amphotericin B
*Candida albicans* ATCC 10231	(−)	35.4 ± 1.2 ^aB^	36.5 ± 1.1 ^aB^	34.6 ± 0.9 ^bB^	34.7 ± 0.4 ^bB^	25.4 ± 0.5 A
*Aspergillus niger* ATCC 16888	(−)	20.8 ± 0.2 ^aB^	21.1 ± 0.4 ^aB^	17.2 ± 0.4 ^bB^	16.9 ± 0.6 ^bB^	23.7 ± 1.2 A

Values are ±SEM (n = 3), means followed by different letters in same row denote significant difference at p < 0.05, paired*-t-*test. Lowercase letters compare means of WEO with SEO, uppercase letters compare means of sample with the standard antibiotic

W1 and W2 are plants collected from Wasita during fall 2017 and 2018, respectively. S1 and S2 are plants collected from Safsaf during fall 2017 and 2018, respectively

Determination of MIL/MLC for the essential oils was performed using broth dilution method (Table 4). The results showed variable effects of the oils against the tested microorganisms. Highest bacteriostatic and bactericidal effects were observed for SEO against *S. aureus*, *P. mirabilis* and *K. pneumonia* with MIC/MLC 0.125/0.25 μg/ml, while lowest activity was for both oils against *P. aeruginosa* and the fungi *A. niger* with MIC/MLC ranging 1- > 1.5 μg/ml. SEO collected during fall 2018 had also better effect against *B. subtilus* with MIC/MLC 0.125/0.25 μg/ml. WEO had better bacteriostatic and bactericidal activity than SEO against *E. coli*, while no significant effect was observed between the activity of both tested samples against *C. albicans*.

**Table 4:** Effect of altitude on the MIC/MLC of the essential oils of *S. thymbra* essential oils obtained from Wasita (WEO) and Safsaf (SEO) localities during two successive years

Test Organisms	Minimum inhibitory and bactericidal concentrations (MIC/MLC, μg/ml^a^)			
	WEO (W1)	WEO (W2)	SEO (S1)	SEO (S2)
*Gram positive organisms*				
*Staphylococcus aureus* ATCC 6538	0.25/0.313	0.188/0.25	0.125/0.25	0.125/0.25
*Bacillus subtilis* ATCC 6051	0.313/0.375	0.25/0.313	0.188/0.25	0.125/0.25
*Gram negative organisms*				
*Escherichia coli* ATCC 8739	0.25/0.25	0.188/0.25	0.25/1	0.25/1
*Pseudomonas aeruginosa* ATCC 9027	1/> 1.5	> 1.5/> 1.5	1/1	1/> 1.5
*Proteus mirabilis* ATCC 7002	0.188/0.25	0.188/0.25	0.125/0.25	0.125/0.25
*Klebsiella pneumoniae* ATCC 13883	0.125/0.375	0.125/0.375	0.125/0.25	0.125/0.25
*Fungi*				
*Candida albicans* ATCC 10231	0.125/0.25	0.125/0.25	0.25/0.25	0.125/0.25
*Aspergillus niger* ATCC 16888	1/1	1/1	1/> 1.5	1//> 1.5

^a^Values are mean resuts of three replicates

W1 and W2 are plants collected from Wasita during fall 2017 and 2018, respectively. S1 and S2 are plants collected from Safsaf during fall 2017 and 2018, respectively

### Effect of altitude on anthelmintic activity of the essential oils

The essential oils collected from cuttings of two successive years from two different altitudes had a strong anthelmintic activity against the tested earthworm which was significantly higher than the standard piperazine citrate used. SEO had a slightly higher anthelmintic activity with shorter time recorded for the paralysis and death of the tested worms (Table 5).

**Table 5:** Effect of altitude on the anthelmintic activity of essential oils of *S. thymbra* obtained from Wasita (WEO) and Safsaf (SEO) localities during two successive years

Test	WEO (W1)	WEO (W2)	SEO (S1)	SEO (S2)	Piperazine citrate (Standard)
	Time for paralysis (min.)				
1% EO	4.32 ± 0.45 ^aB^	4.01 ± 0.57 ^aB^	3.25 ± 0.35 ^bB^	3.03 ± 0.35 ^bB^	9.77 ± 0.78 ^A^
2% EO	3.51 ± 0.52 ^aB^	3.66 ± 0.39 ^aB^	2.15 ± 0.25 ^aB^	2.59 ± 0.05 ^aB^	
3% EO	2.37 ± 0.24 ^aB^	2.66 ± 0.14 ^aB^	1.75 ± 0.15 ^bB^	1.36 ± 0.21 ^bB^	
	Time for death (min.)				
1% EO	6.25 ± 0.66 ^aB^	5.99 ± 0.85 ^aB^	5.58 ± 0.42 ^aB^	5.61 ± 0.36 ^aB^	13.58 ± 0.95 ^A^
2% EO	4.65 ± 0.49 ^aB^	4.14 ± 0.41 ^aB^	4.22 ± 0.22 ^aB^	4.26 ± 0.45 ^aB^	
3% EO	3.89 ± 0.42 ^aB^	3.63 ± 0.43 ^aB^	2.45 ± 0.19 ^bB^	2.41 ± 0.74 ^bB^	

Values are ±SEM (n = 3), means followed by different letters in same row denote significant difference at p < 0.05, paired*-t-*test. Lowercase letters compare means of WEO with SEO, uppercase letters compare means of sample with the standard antibiotic

W1 and W2 are plants collected from Wasita during fall 2017 and 2018, respectively. S1 and S2 are plants collected from Safsaf during fall 2017 and 2018, respectively

### Effect of altitude on cytotoxic activity of the essential oils

**Fig. 1:**
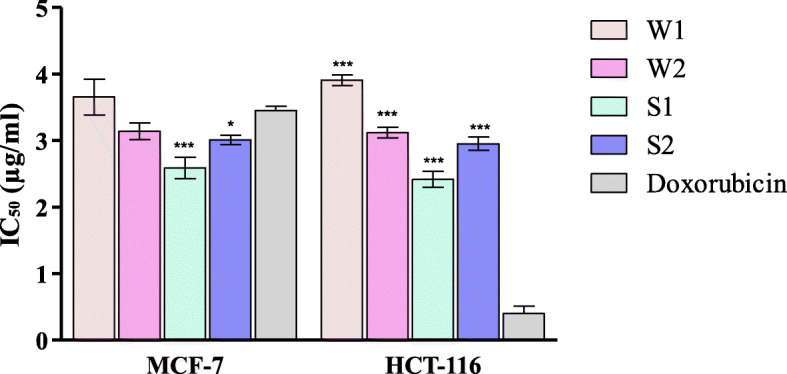
In-vitro cytotoxic activity of S. thymbra oil obtained from Wasita and Safsaf localities on the tested cell lines. Significant differences among means of different treatments were separated using Bonferroni posttests at *P* ≤ 0.05 (*n* = 3) with all treatments compared to the control; doxorubicin. *: *P* < 0.05, ***: *P* < 0.001. W1 and W2 are plants collected from Wasita during fall 2017 and 2018, respectively. S1 andS2 are plants collected from Safsaf during fall 2017 and 2018, respectively

Both WEO and SEO had a strong cytotoxic activity (IC_50_ < 10 μg/ml) on both breast and colon cancer cell lines, with significantly higher activity and better selectivity index observed for SEO (Table 6, Fig. 1).

**Table 6:** Effect of altitude on the cytotoxic activities of the essential oil of *S. thymbra* obtained from Wasita (WEO) and Safsaf (SEO) localities during two successive years

Test	Breast tumor cell line MCF-7	Selectivity index	Colon cancer cell line HCT-116	Selectivity index
	IC_50_ (μg/ml)	IC_50_ (μg/ml)		
Wasita (W1)	3.69 ± 0.51 ^aA^	3.66	3.91 ± 0.14 ^aB^	3.25
Wasita (W2)	3.14 ± 0.22 ^aA^	3.15	3.12 ± 0.14 ^aB^	3.04
Safsaf (S1)	2.75 ± 0.32 ^bB^	4.15	2.42 ± 0.21 ^bB^	4.69
Safsaf (S2)	3.01 ± 0.12 ^bB^	4.82	2.99 ± 0.21 ^bB^	4.78
Doxorubicin	3.45 ± 0.12 ^A^		0.40 ± 0.19 ^A^	

Values are ±SEM (n = 3), means followed by different letters in same column denote significant difference at p < 0.05, paired*-t-*test. Lowercase letters compare means of WEO with SEO, uppercase letters compare means of sample with the standard antibiotic. Selectivity index was calculated as the ratio of the IC_50_ values on V79 cells to those in the tested cancer cell lines. SI > 3 indicates a promising activity

W1 and W2 are plants collected from Wasita during fall 2017 and 2018, respectively. S1 and S2 are plants collected from Safsaf during fall 2017 and 2018, respectively

## Discussion

At higher altitudes**,** average temperatures are lower than those at the base of the mountains. Elevation plays a great role in the health and growth of plants. The type and amount of sunlight, amount of water that plants can absorb and the nutrients that plants receive differ at different altitudes. The main difference observed in the composition of the essential oils was in the major identified compound which was thymol in WEO and carvacrol in SEO. It was also observed that the percentage of total phenolic constituents of WEO is 36.77% while that of SEO is 32.18%. Present results are in agreement with the previously identified compounds in *S. thymbra* essential oil (thymol and carvacrol as well as their precursors; *p*-cymene and γ-terpenene) [8, 43]. Several studies showed that a variation in the essential oil composition occurred in *S. thymbra* plants due to the effect of different factors such as different ecological conditions and even harvesting time [44, 45]. Other studies showed that altitude affects the chemical composition of the essential oils of several plants [31, 46]. Essential oil of *S. hortensis* gave a higher yield of carvacrol at higher altitude [47], which is the case in this present work. Qualitative and quantitative variation in secondary metabolites may be due to several other different environmental, agricultural and genetic factors such as temperature, humidity, amount of rainfall, amount of sunlight, variety of the plant, collection season and even collection timing through the day as well as method of drying and extraction of the plant [13, 34, 48–50]. It is thus interesting to study the composition and biological activity at different geographical areas in order to choose the optimum source of possible drug.

The tested essential oils gave a promising antimicrobial activity against bacteria and fungi. This was evidenced by larger inhibition zones given by the oils compared to the standard antimicrobial agents used, especially against *S. aureus, B. subtilus, E. coli, P. mirabilis, K. pneumonia* as well as *C. albicans*. Values of MIC/MLC were 0.125/0.25 μg/ml for SEO against *S. aureus*, *P. mirabilis* and *K. pneumonia* and for WEO against *B. subtilus*. It was observed that plants growing at lower altitude in Wasita locality had better antifungal activity, while those growing at higher altitude at Safsaf locality had better activity antibacterial activity. Compared with other studies, *S. thymbra* essential oil proved to be an available and natural antimicrobial agent that could be used in food industry to prevent the growth of food borne bacteria or to increase the shelf life of different processed foods [16]. A study showed that the oil of *S. thymbra* showed bacteriostatic activity at 0.001–0.1 mg/ml and was bactericidal at 0.002–0.2 mg/ml and fungistatic effects at 0.001–0.025 mg/ml and fungicidal effects at 0.001–0.1 mg/ml, while the oil main constituents thymol and carvacrol e also showed strong antimicrobial activity and antifungal activity that was even higher than the commercial fungicide bifonazole [8]. The oil is active against certain microbial strains like *Salmonella typhimurium*, *E. coli*, *S. aureus*, *P. aeruginosa*, *C. albicans* and *Moniliophthora perniciosa* [11]. This strong antimicrobial activity may be attributed to the phenolic constituents: thymol and carvacrol which proved to interfere with the cell wall enzymes of bacteria and fungi [51]. Studies also revealed that thymol’s antimicrobial effect, especially against *S. aureus* and *E. coli* could be due to the perturbation of the lipid fraction of the bacterial plasma membrane resulting in the leakage of intracellular materials [52]. Also data show that both carvacrol and thymol had desired antimicrobial effect on *E. coli*, possibly due to their ability to permeabilize and depolarize the cytoplasmic membrane [53]. A study showed that at higher altitudes, plants are exposed to extreme environmental conditions, which stimulate the accumulation of secondary metabolites that causes a further enhancement in the medicinal activity including the antimicrobial effect [54]. Results of the anthelmintic studies augmented that of the antimicrobial results. In literature, the essential oil of *S. thymbra* is well recorded for its anthelmintic, larvicidal, acaricidal and genotoxic activity [3].

Previously, it has been reported that the essential oil of *S. thymbra* possesses a moderate anti proliferative and concentration dependant inhibition of viability of C32 (human amelanotic melanoma cell line) and ACHN (renal cell adenocarcinoma) [6]. Cytotoxicity of the essential oils of other *Satureja* species were evaluated on several cell lines such as J774 macrophage, 5637, KYSE, Fem-X Human Malignant Melanoma, Vero, SW480, MCF7, JET3, A549, THP-1 and HT29/219 [3].

## Conclusion

In conclusion, altitude may influence the chemical composition and biological activity due to a collection of conditions like temperature and climate changes. In this study, *S. thymbra* plants collected during two successive years, growing at a lower altitude gave a better essential oil yield, higher yield of thymol as well as better antifungal activity. However, plants growing at higher altitudes gave better yield of carvacrol, improved antibacterial, anthelmintic activity and better cytotoxic activity against colon and breast cancer cell lines. Therefore, altitude at which the plant grows should be considered according to its intended use. Moreover, further studies on samples collected from more altitudes, as well studying the effect of other different environmental factors at these altitudes, such as temperature, humidity and amount of sunlight, should be employed in order to augment the results of this study.

## Data Availability

All obtained data have been included into the manuscript.

## Abbreviations

*A. niger*: *Aspergillus niger*
ACHN: Renal cell adenocarcinoma
AT: Identification based on co-chromatography with authentic samples
ATCC: American Type Culture Collection
*B. subtilus*: *Bacillus subtilis*
BHI: Brain heart infusion
*C. albicans*: *Candida albicans*
C32: Human amelanotic melanoma cell lines
CFU: Colony forming unit
CSB: Casein soy broth
DMSO: Dimethyl sulfoxide
*E. coli*: *Escherichia coli*
GC/MS: Gas chromatography/mass spectrometry
GC-FID: Gas chromatography/flame ionization detection
HCT-116: Human colon carcinoma cell lines
hrs.: Hours
HSV-1: Hepres simplex virus 1
IC_50_: Half maximal inhibitory concentration
*K. pneumonia*: *Klebsiella pneumoniae*
MCF-7: Human breast cancer cell lines
MIC/MLC: Minimum inhibitory and lethal concentrations
*P. aeruginosa*: *Pseudomonas aeruginosa*
*P. mirabilis*: *Proteus mirabilis*
PCA: Plate count agar
RI: Retention index
S1: Plants collected from Safsaf during fall 2017
S2: Plants collected from Safsaf during fall 2018
*S. aureus*: *Staphylococcus aureus*
*S. fortii*: *Satureja fortii*
*S. hortensis*: *Satureja hortensis*
*S. maltophilia*: *Stenotrophomonas maltophilia*
*S. thymbra*: *Satureja thymbra*
SARS-CoV: Severe acute respiratory syndrome coronavirus
SDA: Sabouraud dextrose agar
SDB: Sabouraud Dextrose Broth
SEM: Standard error mean
SEO: Safsaf essential oil
SI: Selectivity index
SRB: Sulforhodamine B stain
TSA: Trypticase soy agar
v/v: Volume/volume
V79: Normal hamster lung fibroblasts
W1: Plants collected from Wasita during fall 2017
W2: Plants collected fromWasita during fall 2018
WEO: Wasita essential oil
